# Unraveling Heart Inflammation: Recurrent Myopericarditis Caused by Coxsackie A Virus—A Case Report

**DOI:** 10.1002/ccr3.9630

**Published:** 2024-11-27

**Authors:** Maisha Maliha, Vikyath Satish, Kuan Yu Chi, Nishat Shama, Sananda Halder, Sumaiya Monjur Oishy, Amrin Kharawala, Dimitrios Varrias, Robert T. Fallaice

**Affiliations:** ^1^ Dhaka Medical College Dhaka Bangladesh; ^2^ Jacobi Medical Center New York USA; ^3^ BIRDEM Dhaka Bangladesh

**Keywords:** Coxsackie, myocarditis, myopericarditis, pericarditis, recurrent

## Abstract

Acute recurrent myopericarditis, characterized by the occurrence of a new myopericarditis event following a symptom‐free interval of 4–6 weeks, is relatively rare lacking definitive guidelines for management. Understanding its prevalence, causes, and optimal management is challenging due to limited data and insufficient guidelines. This case outlines the diagnostic work‐up and different management modalities for recurrent myopericarditis. A 44‐year‐old African American man with a past medical history of myopericarditis a year ago presented with fever and chest pain for 4 days. The patient was found to have elevated troponin, CRP, and ESR; pericardial effusion along with a Coxsackie A virus titer of 1:800, suggestive of Coxsackie A virus–induced recurrent myopericarditis. The patient responded well to colchicine and a tapering dose of ibuprofen, achieving significant resolution in the pericardial effusion. Recurrent myopericarditis caused by Coxsackie A virus is a relatively rare phenomenon. New onset myopericarditis can be caused by various factors such as infections, autoimmune disorders, neoplasms, metabolic issues, trauma, and drugs, with recurrence rates of 15%–50% in pericarditis patients. Coxsackie A virus is an important and rare etiology of recurrent myopericarditis due to its unique immune evasive traits. The treatment modalities guided by definitive guidelines for recurrent pericarditis can be applied in recurrent myopericarditis with significant resolution of symptoms. Although there are no specific guidelines for managing recurrent myopericarditis, using approaches designed for recurrent pericarditis has shown promising results, and the immune evasive nature of Coxsackie A virus underscores the need for further research to improve our understanding and treatment of this condition.

## Graphical abstract

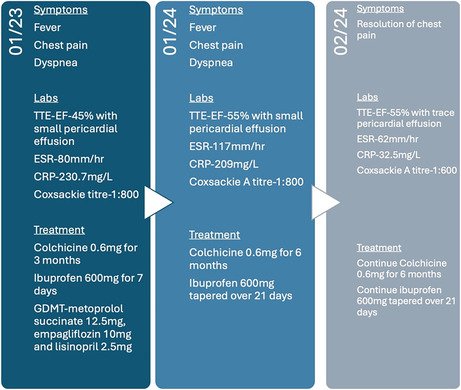

**Summary**

- Acute recurrent myopericarditis caused by Coxsackie A virus is rare and lacks definitive guidelines, posing diagnostic and treatment challenges.
- While management strategies for recurrent pericarditis utilized in recurrent myopericarditis show promise, Coxsackie A virus's immune evasion underscores the need for further research.

## 1 Introduction

Myopericarditis, according to 2015 European Society of Cardiology (ESC) guidelines, is characterized by predominant pericarditis with myocardial involvement and can be clinically confirmed in patients meeting definite criteria for acute pericarditis, alongside elevated biomarkers of myocardial injury (troponin I or T, CK‐MB) [1, 2], This diagnosis is established in the absence of newly developed impaired left ventricular function in echocardiography or Cardiovascular Magnetic Resonance (CMR) [2]. While pericarditis is found to be the cause of 0.1% of hospitalizations and myocarditis occurred in 17 cases per 100,000 persons in a study involving military recruits, the exact incidence of myopericarditis remains unknown [3, 4].

Initial manifestations of myopericarditis can include precordial positional chest pain, fever fatigue, dyspnea, and palpitations [5]. It can be divided into infectious and non‐infectious etiologies, as shown in Table 1 [6]. Among these, Coxsackie B virus is found in 50% of patients with newly identified cases of myopericarditis [7]. However, it is very rare to identify Coxsackie A virus as a cause of either initial or recurrent myopericarditis [8, 9]. Furthermore, while recurrent pericarditis affects 15%–50% of individuals with pericarditis in the United States, amounting to around 20,000 people per year, the prevalence of recurrent myopericarditis is not clearly defined [10, 11, 12]. We present the case of a 44‐year‐old man with recurrent myopericarditis due to Coxsackie A virus who was treated with colchicine and tapering dose of ibuprofen.

**TABLE 1:** Common etiologies of new onset of myopericarditis.

Infectious causes
Viral: Coxsackievirus, echovirus, herpes virus, adenovirus, parvovirus B19
Bacteria: *Mycobacterium tuberculosis* , *Coxiella burnetii* , *Borrelia burgdorferi*
Fungal: Histoplasma, Aspergillus, Candida, Blastomyces
Autoimmune
Systemic lupus erythematosus, rheumatoid arthritis, sarcoidosis, systemic vasculitis
Neoplastic
Primary cardiac tumor
Secondary metastatic tumors, commonly lung and breast cancer
Metabolic
Hypothyroidism, Uremia
Trauma
Post‐radiation, post pericardiectomy syndrome
Drug‐related
Procainamide, hydralazine, doxorubicin, danorubicin, cyclophosphamide
Vaccine
SARS‐Cov‐2

## 2 Case History

A 44‐year‐old male with a history of myopericarditis 1 year ago initially came to the emergency department with fever and chest pain for 4 days. The patient presented with 8/10 non‐exertional pressure‐like chest pain localized to the mid‐sternal region. The pain was described as radiating to the left shoulder and was alleviated by leaning forward and taking ibuprofen. He did not experience dyspnea, orthopnea, paroxysmal nocturnal dyspnea, changes in exercise tolerance, palpitations, diaphoresis, flu‐like symptoms, sore throat, joint pain, or rash. He had no recent travel history or exposure to sick contacts. His past medical history included non‐ischemic cardiomyopathy, sickle cell trait, and latent tuberculosis.

The patient had a temperature of 100.9°F (38.3°C) and remained hemodynamically stable. On examination, he had trace bilateral edema, with a clear lung exam and normal heart sounds without any murmur.

This presentation closely resembled his previous admission a year ago, where the patient experienced fever, positional chest pain, and dyspnea on exertion and was subsequently diagnosed with Coxsackie A virus–induced myopericarditis and pleuritis. In that admission, the patient was also found to have pleural effusion, pericardial effusion without tamponade physiology, along with non‐ischemic cardiomyopathy with an ejection fraction (EF) of 45%. In that hospitalization, the patient was started on colchicine 0.6 mg per day for 3 months and ibuprofen 600 mg three times a day for 7 days for myopericarditis with subsequent resolution of symptoms. He was also started on metoprolol succinate 12.5 mg, lisinopril 2.5 mg, and empagliflozin 10 mg for his cardiomyopathy. He was followed up at 3, 5, and 12 months, during which he remained symptom‐free from myopericarditis. Inflammatory markers, including erythrocyte sedimentation rate (ESR), C‐reactive protein (CRP), and high‐sensitivity (hs)‐troponin, decreased. His Coxsackie A virus titer dropped from 1:800 initially to 1:600 at 3 months and eventually became undetectable. Additionally, his EF improved to 55% at the 12‐month follow‐up.

Furthermore, the patient's latent tuberculosis was treated with 6 months of isoniazid monotherapy prior to his first episode of myopericarditis.

## 3 Methods

### 3.1 Investigations

Significant laboratory findings included an initial elevation in hs‐ troponin to 34 ng/dL (*n*: 0–22 ng/dL). The patient was also found to have elevated inflammatory markers with ESR of 117 mm/h (*n*: 0–15 mm/h) and CRP of 209 mg/L (*n*: 0–5 mg/L). Coxsackie A virus titer Immunoglobulin (Ig) M was 1:800 (*n*: 1:100) for serotypes A‐7, A‐9, A‐16, and A‐24 detected by indirect fluorescent antibody. Coxsackie B virus IgM titers were within normal range.

**FIGURE 1:**
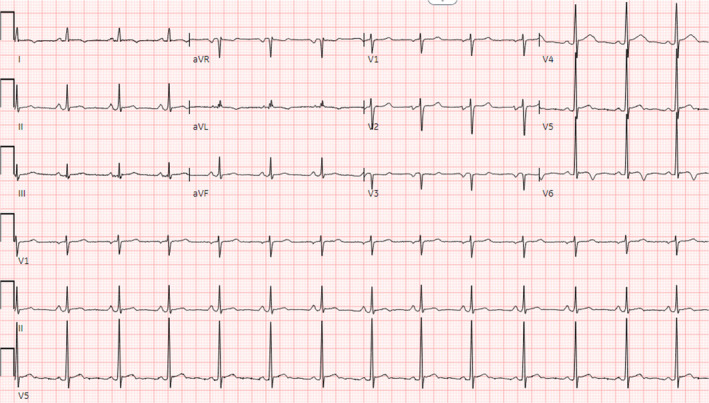
EKG with sinus rhythm and non‐specific T wave inversions.

**FIGURE 2:**
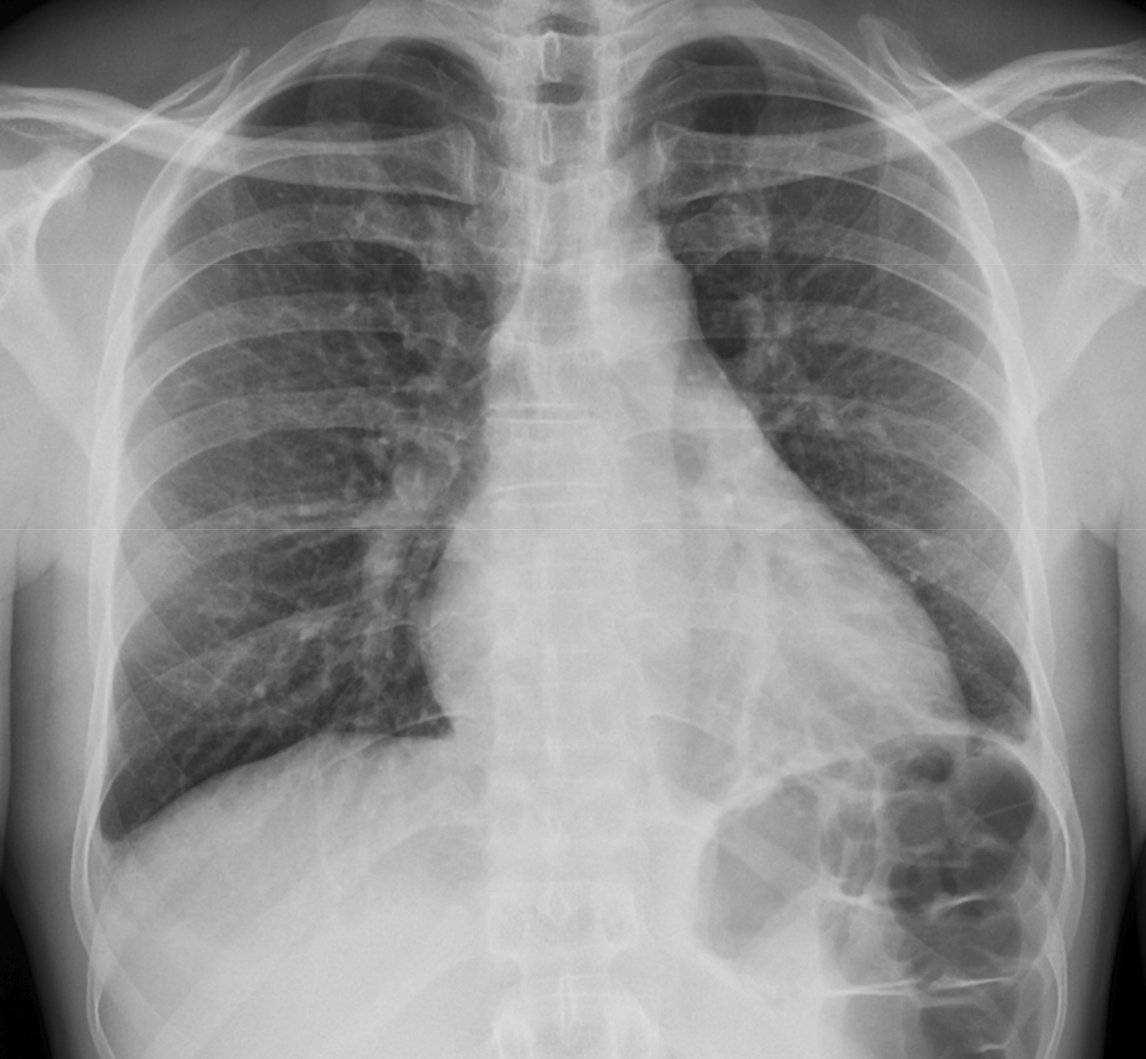
Chest X‐ray showing bilateral small pleural effusions.

**FIGURE 3:**
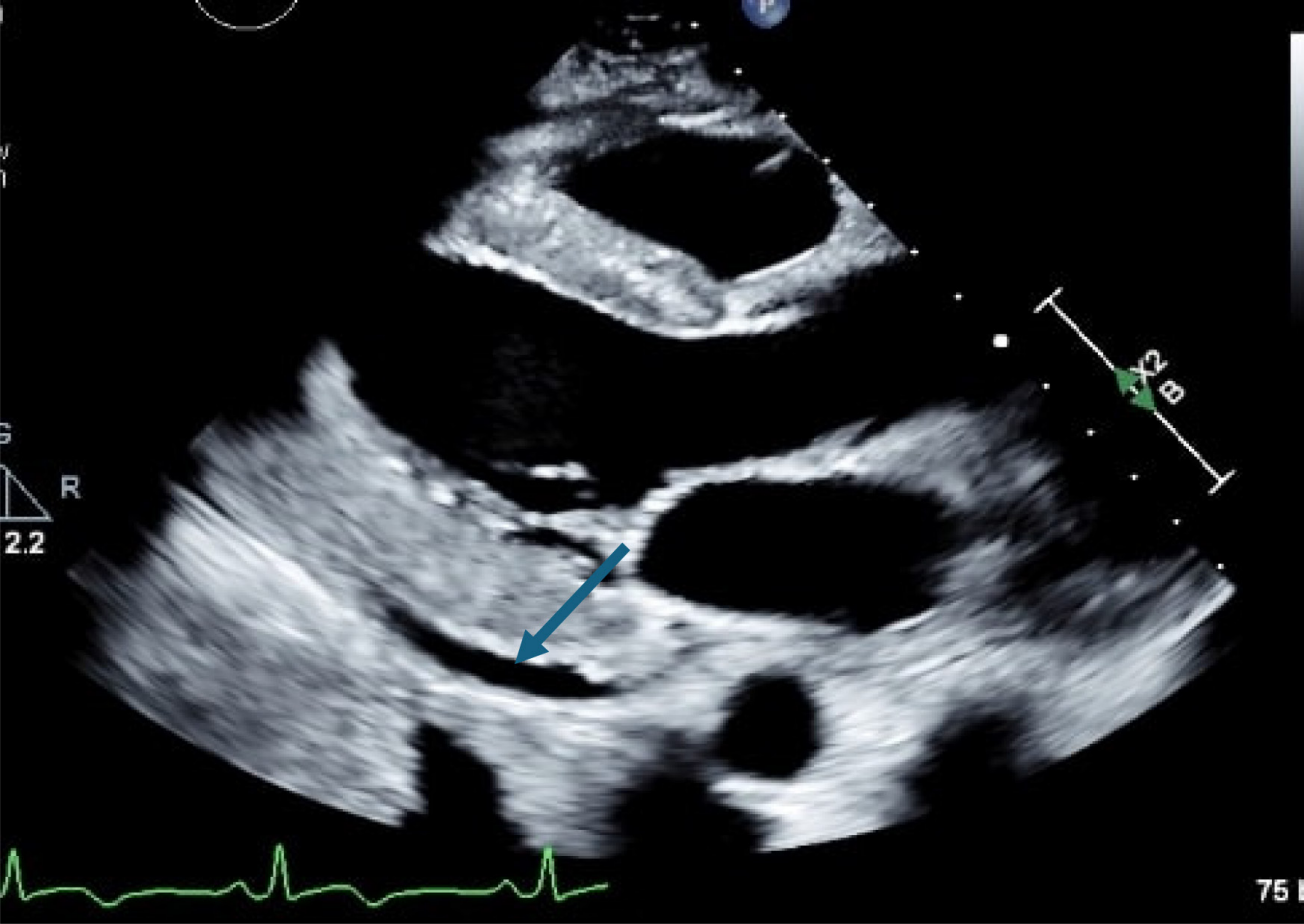
TTE. Para‐sternal long axis view showing pericardial effusion of < 1 cm (blue arrow) on initial presentation.

The electrocardiogram (EKG) showed sinus rhythm with non‐specific T wave inversions without significant ST elevations or PR depressions, as shown in Figure 1. Bilateral small pleural effusions were noted on the chest X‐ray, as shown in Figure 2. Transthoracic echocardiogram (TTE) showed a small pericardial effusion of less than 1 cm in size, without respiratory variation in transaortic and trans tricuspid flow, and no indications of tamponade physiology, as shown in Figure 3. However, confirmation of the diagnosis of myocarditis by endomyocardial biopsy was not done as, according to 2015 ESC guidelines, myopericarditis with no symptoms of acute decompensated heart failure does not clinically require endomyocardial biopsy [2].

Further investigations to rule out other etiologies, which included antinuclear antibody, rheumatoid factor, thyroid‐stimulating hormone (TSH) level, acid‐fast bacilli sputum, antibody levels of viruses including Epstein Barr virus, herpes virus, parvovirus, influenza, hepatitis C, severe acute respiratory syndrome coronavirus 2 (SARS‐CoV‐2), and adenovirus, were negative. This effectively ruled out autoimmune, endocrine, malignant, and infectious etiologies other than Coxsackie A virus for myopericarditis.

### 3.2 Differential Diagnosis

Initial potential differential diagnoses considered were recurrent myopericarditis, acute coronary syndrome (ACS), pneumonia, and pulmonary embolism. As per 2015 ESC guidelines [2], the patient tested positive for two out of four criteria for pericarditis: characteristic chest pain of pericarditis and the presence of pericardial effusion observed on TTE, along with a new event following a symptom‐free interval of 4–6 weeks for recurrence and elevated troponin for myocarditis. Subsequently, the patient was diagnosed with Coxsackie virus A–induced recurrent myopericarditis.

### 3.3 Treatment

Implementing 2015 ESC guidelines for recurrent pericarditis [2] to recurrent myopericarditis, the patient was initiated on a regimen of colchicine 0.6 mg twice per day for a duration of 6 months and a tapering dose of ibuprofen starting at 600 mg three times daily for 7 days, followed by 600 mg twice daily for 7 days, and finally 600 mg once daily for 7 days, completing a total treatment duration of 21 days.

## 4 Results

**FIGURE 4:**
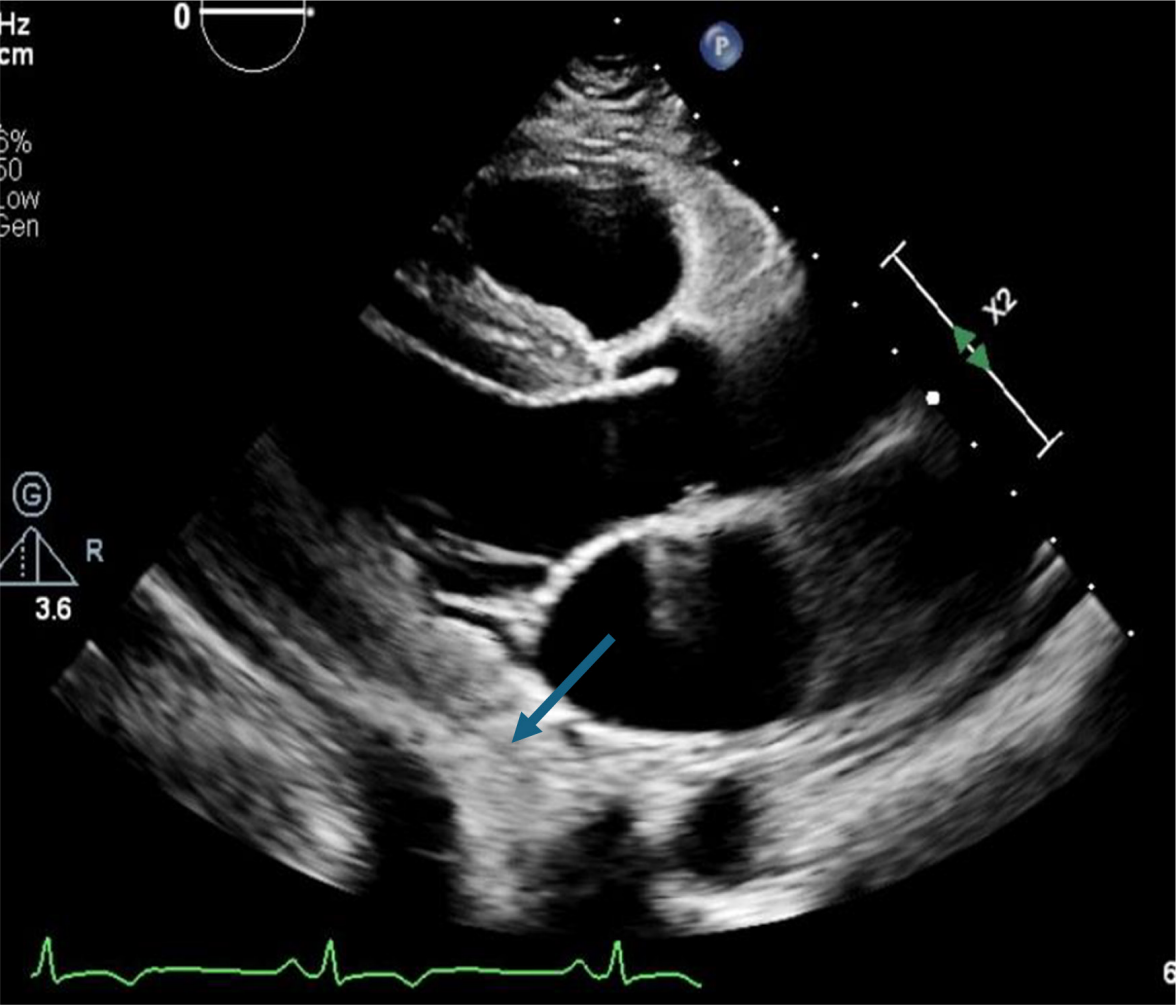
TTE. Parasternal long axis view showing decrease in size of pericardial effusion to trace(arrow) after treatment in follow up.

**FIGURE 5:**
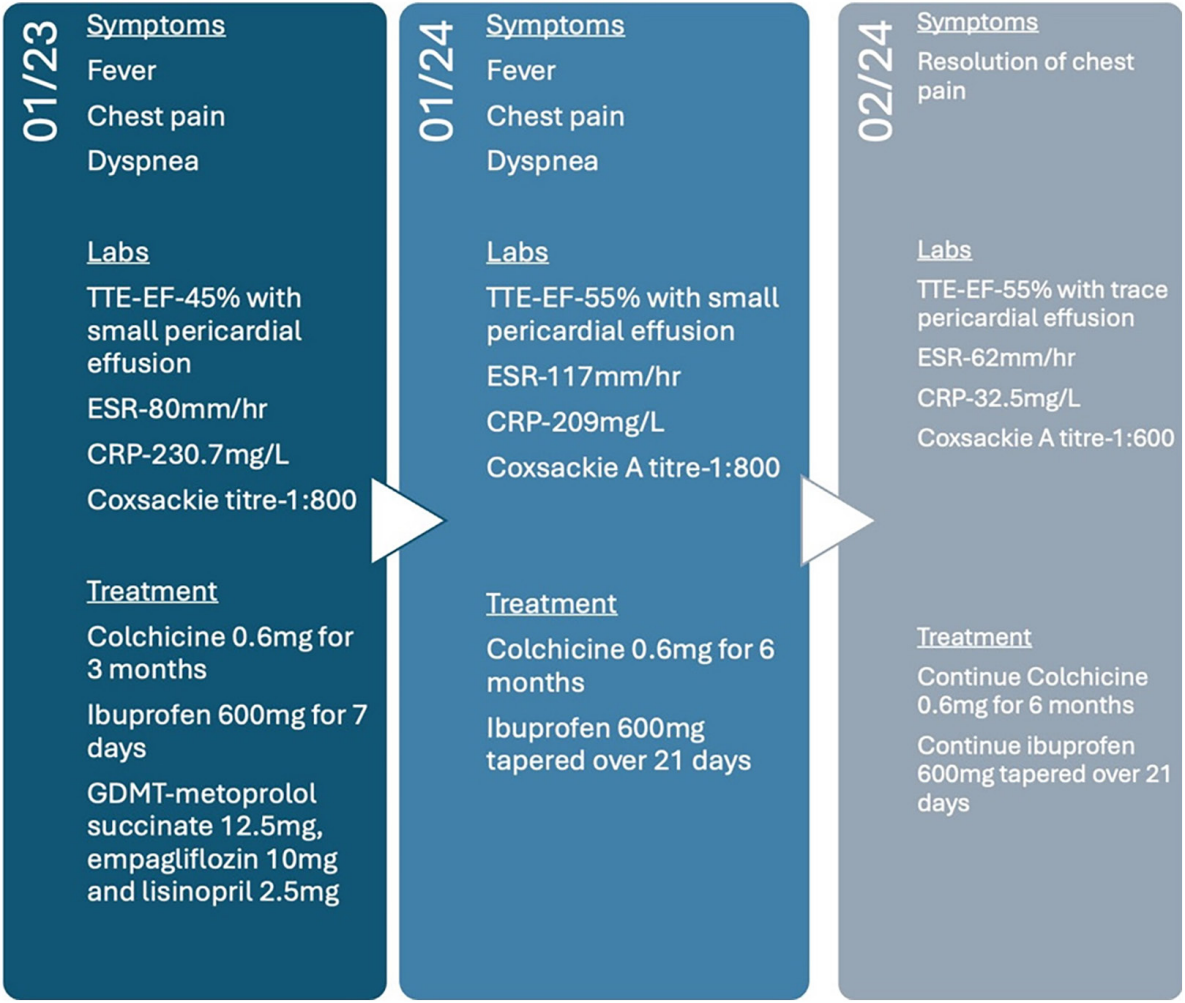
A summary of the clinical presentation.

The patient's chest pain significantly improved, and the inflammatory markers, ESR (62 mm/h), CRP (33.5 mg/L), and hs‐troponin (22 ng/dL) showed a decreasing trend over 14 days. The patient was discharged with an outpatient cardiology follow up. At the follow‐up visit 4 weeks later, the chest pain had completely resolved, and a notable reduction in pericardial effusion size (from less than 1 cm to trace) was observed in the repeat TTE, as shown in Figure 4. The Coxsackie IgM titer decreased to 1:600. The case summary is summarized in Figure 5.

## 5 Discussion

We present a case of recurrent myopericarditis due to Coxsackie A virus. Coxsackie A virus is a rare factor that causes recurring myopericarditis, and there are no specific guidelines for treating it. However, using strategies meant for dealing with recurring pericarditis has shown promise in achieving good results. The way Coxsackie A virus avoids the immune system is a key reason for its ability to cause repeat episodes of myopericarditis, emphasizing the importance of more research to understand this process better and potentially improve treatment methods.

There are multiple etiologies for new onset of myopericarditis, which include infectious, autoimmune, neoplastic, metabolic, trauma, drug‐related, and other causes, as outlined in Table 1 [1]. Among the various causes of myopericarditis, tuberculosis is a rare etiology, accounting for only 0.14%–2% of cases in tuberculosis patients [13, 14]. Most cases of tuberculous myopericarditis are asymptomatic; however, some may present with conduction abnormalities, including atrioventricular block and ventricular arrythmias [15]. There is no evidence or study available to suggest that latent tuberculosis can lead to the progression or recurrence of myopericarditis. Additionally, given that the patient received appropriate treatment for latent tuberculosis and no acid‐fast bacilli were found in the sputum during this hospitalization, it can be safely concluded that tuberculosis was not the cause of his recurrent myopericarditis. Instead, Coxsackie A virus has been identified as the likely inciting factor. Furthermore, there is limited data available regarding recurrent myopericarditis, and it is typically rare to identify Coxsackie A virus as the culprit, adding uniqueness to this case.

According to 2015 ESC guidelines, the treatment of recurrent pericarditis primarily includes colchicine 0.5 mg for 6 months, aspirin, or non‐steroidal inflammatory drugs (NSAIDs) (Class I, Level A recommendation) [2]. Corticosteroids are not recommended as the initial treatment (Class III, Level B), and the duration and response to treatment should be guided using CRP as a marker (Class IIa, Level C) [2].

The second line modalities of treatment include azathioprine [16], IVIG [17], and IL‐1 receptor antagonists such as Anankira [18] and Rilonacept [19] (Class IIb, Level C) [2]. Pericardiectomy should be contemplated as a final resort for individuals experiencing chronic constriction or pericarditis with symptoms that cannot be effectively treated [20]. The treatment modalities are summarized in Table 2. Few case reports [18, 21, 22, 23, 24] highlight the effectiveness of colchicine and indomethacin in recurrent myopericarditis; however, currently, there are no established guidelines for managing recurrent myopericarditis or determining the duration of treatment. In our case, we followed the ESC guidelines for managing recurrent pericarditis, adapting them to recurrent myopericarditis. We administered colchicine for 6 months and ibuprofen for 21 days, aligning with the ESC recommendation for recurrent pericarditis, and observed promising results.

**TABLE 2:** Summary of ESC guidelines for recurrent pericarditis.

ESC recommendation	Class	Level
Colchicine	I	A
NSAIDS or aspirin	I	A
Azathioprine	IIb	C
IVIG	IIb	C
IL‐1 receptor antagonist‐Anankira, Rilonacept	IIb	C
Corticosteroids are not recommended as first line therapy	III	B

Coxsackie A primarily causes mild, self‐limiting diseases involving the skin and mucous membranes [25, 26]. In contrast, Coxsackie B is more likely to cause severe systemic diseases, including myocarditis, pericarditis, and pancreatitis [25, 26]. Coxsackievirus B is believed to play a role in approximately 25%–40% of initial instances of acute myopericarditis and dilated cardiomyopathy [27]. There is limited data on the prevalence of Coxsackie A virus–induced recurrent myopericarditis, and the pathophysiology is poorly understood. One possible hypothesis is that both Coxsackie A and B viruses can evade the immune system and lead to recurrent myocardial and pericardial damage [28]. Both cell surface and internal Toll‐like receptors (TLR) have been implicated in the immune response to Coxsackie A virus [28]. Furthermore, Coxsackie virus leads to impaired apoptosis by CD8+T cells by having an inhibitory effect on antigen presentation via the MHC Class I pathway [29]. These interactions of Coxsackie virus with the immune system have led to long‐term persistent infection in a variety of cell types, including human myocardial cells [30, 31]. Coxsackievirus‐neutralizing IgM antibodies typically appear 3 days after infection, peak around day seven, and usually diminish by 3 months [32]. However, in this patient, the Coxsackie A IgM titer began to decline earlier, at the 1‐month follow‐up. This faster loss of immunity against Coxsackie A virus may have contributed to recurrent episodes of myopericarditis. However, additional studies are needed to understand better how the Coxsackie A virus evades the immune system and causes recurrent myopericarditis.

Hence, Coxsackie A virus should always be considered an important etiology in recurrent myopericarditis, and treatment approaches established for recurrent pericarditis can be employed in managing recurrent myopericarditis.

## Author Contributions

**Maisha Maliha:** resources, software, supervision, visualization, writing – original draft, writing – review and editing. **Vikyath Satish:** resources, software, visualization, writing – original draft, writing – review and editing. **Kuan Yu Chi:** writing – original draft, writing – review and editing. **Nishat Shama:** writing – original draft, writing – review and editing. **Sananda Halder:** software, writing – original draft, writing – review and editing. **Sumaiya Monjur Oishy:** software, writing – review and editing. **Amrin Kharawala:** writing – review and editing. **Dimitrios Varrias:** writing – review and editing. **Robert T. Fallaice:** supervision.

## Ethics Statement

This case report adheres to ethical guidelines by ensuring patient confidentiality, obtaining informed consent, and prioritizing the welfare and rights of the patient throughout the study.

## Consent

In adherence to stringent ethical standards, I affirm that the research obtained informed written consent from participant, ensured data confidentiality without patient identifiers, and maintained transparency and objectivity throughout.

## Conflicts of Interest

The authors declare no conflicts of interest.

## Data Availability

The authors have nothing to report.
